# Sensory irritation as a basis for setting occupational exposure limits

**DOI:** 10.1007/s00204-014-1346-z

**Published:** 2014-09-03

**Authors:** Thomas Brüning, Rüdiger Bartsch, Hermann Maximillian Bolt, Herbert Desel, Hans Drexler, Ursula Gundert-Remy, Andrea Hartwig, Rudolf Jäckh, Edgar Leibold, Dirk Pallapies, Albert W. Rettenmeier, Gerhard Schlüter, Gisela Stropp, Kirsten Sucker, Gerhard Triebig, Götz Westphal, Christoph van Thriel

**Affiliations:** 1 Institute for Prevention and Occupational Medicine of the German Social Accident Insurance (IPA), Bochum, Germany; 2 Permanent Senate Commission for the Investigation of Health Hazards of Chemical Compounds in the Work Area (MAK Commission), TU Munich, Hohenbachernstr. 15-17, 85350 Freising-Weihenstephan, Germany; 3 Leibniz Research Centre for Working Environment and Human Factors, TU Dortmund, Ardeystr. 67, 44139 Dortmund, Germany; 4 University Medical Center Göttingen, Georg-August-University, GIZ-Nord Poisons Center, Forensic and Clinical Toxicology Lab, Robert-Koch-Str. 40, 37075 Göttingen, Germany; 5 Institute and Outpatient Clinic of Occupational, Social and Environmental Medicine, University of Erlangen-Nuremberg (IPASUM), Schillerstr. 25/29, 91054 Erlangen, Germany; 6 Federal Institute for Risk Assessment (BfR), Thielallee 88-92, 14195 Berlin, Germany; 7 Department of Food Chemistry and Toxicology, Karlsruhe Institute of Technology (KIT), Institute of Applied Biosciences, Adenauerring 20, 76131 Karlsruhe, Germany; 8 BASF SE, 67056 Ludwigshafen, Germany; 9 Institute for Medical Informatics, Biometry and Epidemiology (IMIBE) of the University of Essen, Hufelandstr. 55, 45122 Essen, Germany; 10 Bayer Pharma AG, Global Drug Discovery - Global Early Development – Product Stewardship Industrial Chemicals & Operations Research Center, Aprather Weg, Building 514, 42096 Wuppertal, Germany; 11 Institute and Outpatient Clinic for Occupational and Social Medicine, University Heidelberg, Voßstr. 2, 69115 Heidelberg, Germany

**Keywords:** Local irritants, Chemosensory perception, Regulatory toxicology, Interspecies extrapolation

## Abstract

There is a need of guidance on how local irritancy data should be incorporated into risk assessment procedures, particularly with respect to the derivation of occupational exposure limits (OELs). Therefore, a board of experts from German committees in charge of the derivation of OELs discussed the major challenges of this particular end point for regulatory toxicology. As a result, this overview deals with the question of integrating results of local toxicity at the eyes and the upper respiratory tract (URT). Part 1 describes the morphology and physiology of the relevant target sites, i.e., the outer eye, nasal cavity, and larynx/pharynx in humans. Special emphasis is placed on sensory innervation, species differences between humans and rodents, and possible effects of obnoxious odor in humans. Based on this physiological basis, Part 2 describes a conceptual model for the causation of adverse health effects at these targets that is composed of two pathways. The first, “sensory irritation” pathway is initiated by the interaction of local irritants with receptors of the nervous system (e.g., trigeminal nerve endings) and a downstream cascade of reflexes and defense mechanisms (e.g., eyeblinks, coughing). While the first stages of this pathway are thought to be completely reversible, high or prolonged exposure can lead to neurogenic inflammation and subsequently tissue damage. The second, “tissue irritation” pathway starts with the interaction of the local irritant with the epithelial cell layers of the eyes and the URT. Adaptive changes are the first response on that pathway followed by inflammation and irreversible damages. Regardless of these initial steps, at high concentrations and prolonged exposures, the two pathways converge to the adverse effect of morphologically and biochemically ascertainable changes. Experimental exposure studies with human volunteers provide the empirical basis for effects along the sensory irritation pathway and thus, “sensory NOAEC_human_” can be derived. In contrast, inhalation studies with rodents investigate the second pathway that yields an “irritative NOAEC_animal_.” Usually the data for both pathways is not available and extrapolation across species is necessary. Part 3 comprises an empirical approach for the derivation of a default factor for interspecies differences. Therefore, from those substances under discussion in German scientific and regulatory bodies, 19 substances were identified known to be human irritants with available human and animal data. The evaluation started with three substances: ethyl acrylate, formaldehyde, and methyl methacrylate. For these substances, appropriate chronic animal and a controlled human exposure studies were available. The comparison of the sensory NOAEC_human_ with the irritative NOAEC_animal_ (chronic) resulted in an interspecies extrapolation factor (iEF) of 3 for extrapolating animal data concerning local sensory irritating effects. The adequacy of this iEF was confirmed by its application to additional substances with lower data density (acetaldehyde, ammonia, *n*-butyl acetate, hydrogen sulfide, and 2-ethylhexanol). Thus, extrapolating from animal studies, an iEF of 3 should be applied for local sensory irritants without reliable human data, unless individual data argue for a substance-specific approach.

## Scope

Compiling toxicological profiles for chemicals in the workplace demonstrate that sensory irritation often appears to be a very sensitive and relevant end point in human risk assessment. Accordingly, 40 % of the occupational exposure limit values (OELs) are based on the avoidance of sensory irritation (Dick and Ahlers 1998; Edling and Lundberg 2000; van Thriel et al. 2006b). This end point is related to the interaction of volatile chemicals with neuronal sensors located in mucous membranes of the respiratory tract (RT) and the eyes. In many cases, data from controlled human studies are either not available or inadequate, so OELs are predominantly derived from animal data investigating local effects in the RT. These effects are usually measured as tissue irritation. Taking these different end points (sensory vs. tissue irritation) and the intra- and interspecies differences into consideration, the application of default factors is required to establish an OEL based on animal data. This overview provides a process-oriented proposal for the derivation of threshold limit values for substances causing local effects that was developed by a Joint Working Group of the MAK Commission of the German Research Foundation (DFG) and the Subcommittee III of the Committee for Hazardous Substances (AGS) of the Federal Ministry of Labor and Social Affairs. The present paper will exclusively focus on substances/substance classes for which sensory or tissue irritation of the eyes and/or of the upper respiratory tract (URT) (i.e., nose, pharynx, and larynx) has been identified as the most sensitive end points. OEL setting for substances exerting their most sensitive adverse effect in the lower respiratory tract (LRT) is accomplished according to the usual proceedings by using standard default factors.

Within this conceptual framework, it is mandatory that human data will not be used without considering animal data (a) to form a full picture of the mode of actions and (b) to take into account the thresholds of the relevant effects observed in the LRT of animals. Even if there are indications that a chemical might cause sensory irritation, the OEL setting process still requires a full review of *all* available toxicity data of the substance in question. In a “case-by-case” approach, all relevant information from human, animal, and other experimental studies as well as background data are collected. Subsequently, the adverse effect(s) considered being crucial for the setting of an OEL is/are established. In addition, the physicochemical properties (e.g., water solubility, reactivity, electrophilicity) of a compound can provide information regarding local effects at the various target sites of the upper or LRT.

The present paper provides a brief description of the physiology of the relevant target sites, a conceptual model of the two modes of action underlying local effects in the URT and the eyes, and finally an empirical approach, based on well-studied model compounds, for the derivation of a default factor for interspecies differences.

According to the proposed model, a “sensory NOAEC_human_” can be derived from reflexes and defense mechanisms caused by stimulation of peripheral nerves (e.g., trigeminal nerve endings) that can be measured distortion free by means of physiological parameters (e.g., eyeblink frequency). These effects are based on neuronal activation and do not represent adverse end points per se. However, if these sensory-mediated defense mechanisms/reflexes are elicited continuously under conditions of higher or prolonged exposure that impede reversibility, they can finally result in adverse health effects (see Fig. 3b). Hence, if an OEL for sensory irritation is derived from such a solid, sensory NOAEC_human_ and interindividual variation can be taken into account statically (e.g., by applying a benchmark dose calculation of the BMCL), a general intraspecies default factor must not be adopted for OEL setting.

## Part 1: Local effects of irritants on the upper respiratory tract and the mucous membranes of the eyes in the working environment—basic physiological principles and a glossary of terminology

### Introduction

A standard textbook in toxicology provides the following distinction between systemic and local effects: “Local effects refer to those that occur at the site of first contact between the biological system and the toxicant” (Klaassen 2008).

In the working environment, chemicals can be present in the vapor phase but also as aerosols (i.e., liquid droplets in a gas), and thus, inhalation is the major route of exposure for many compounds. Consequently, for the quantitative risk assessment of workplace chemicals, it can be assumed that the dominant effects caused by locally acting chemicals are biochemical and morphological alterations of the RT. Moreover, chemicals can interact with the ocular surface, composed of cornea and conjunctiva that is covered by the tear film. Regardless of active inhalation or passive diffusion, chemicals can also stimulate the sensory sentinels located in the RT (e.g., olfaction or trigeminal chemoreception in the nasal cavity) and on the ocular surface and, at higher concentrations, may elicit reflexes and immunological defense mechanisms.

The physicochemical properties of a chemical such as molecular size and structure, volatility, water solubility, reactivity, and lipophilicity (i.e., log*P*_ow_) determine to a large extent the route of exposure (e.g., inhalation), the target site (e.g., upper or LRT), and the transport into aqueous biofluids (e.g., nasal mucous, tear film) and across lipid membranes. For instance, lipophilic or surface-active lipophilic compounds may cause a destabilization of the eye tear film leading to epithelial damage of the conjunctiva (Wolkoff et al. 2003).

Aerosols are deposited in the RT according to their aerodynamic diameter. Aerosols with a mass median aerodynamic diameter (MMAD) below ca. 100 µm can be inhaled, aerosols below 10 µm can enter the smaller airways, and aerosols with an MMAD below ca. 4 µm can reach the alveoli (CEN 1993).

The following sections describe the physiological properties of the various compartments of the RT and the eyes, as well as their sensory innervation.

### Morphology and physiology of the target sites

Roughly, the RT can be divided into the upper (URT) and the LRT. The URT consists of nose, pharynx, and larynx. All segments below the trachea are considered to compose the LRT. In detail, these are bronchi, bronchioles, and alveoli. Mucosal membranes of different morphology and histology cover the entire URT as well as bronchi and bronchioles. The lung epithelial lining fluid (ELF), also called surfactant, a watery layer containing a complex mixture of proteins, phospholipids and fatty acids, protects the alveoli (Shelley et al. 1984; Wright and Clementis 1987; Gharib et al. 2010). The ELF prevents alveolar collapse and preserves bronchiolar patency during respiration and is involved in the protection of the lung from injuries and infection (Griese 1999). During inhalation, volatile chemicals are actively transported to these various compartments of the RT. The ocular surface is continuously exposed to volatile chemicals and diffusion into the tear film covering the outer eye causes local effects described and perceived as eye irritation (Wolkoff et al. 2003).

**Fig. 1:**
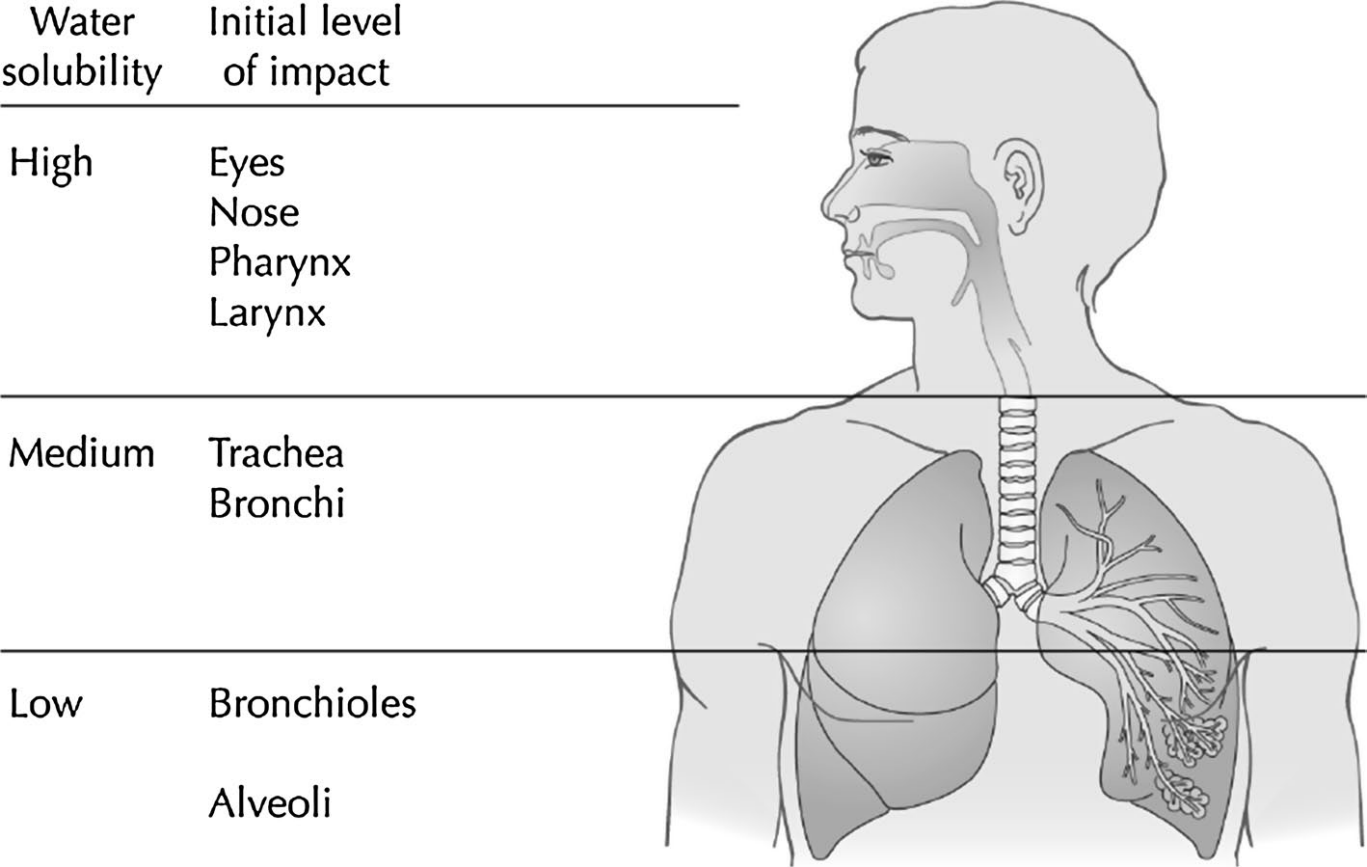
Association between water solubility and predominate effect site of volatile chemicals (cited from Shusterman et al. 2003)

As mentioned earlier, the anatomical region within the RT where a particular compound preferably deposits is partly determined by its water solubility (Shusterman 2003). Figure 1 illustrates this association. In addition to water solubility, reactivity and dosage are important factors affecting processes such as diffusion and deposition in the various compartments of the RT/the ocular surface. In humans, the ratio between nasal and oral breathing might also influence the first contact site of an inhaled chemical within the RT.

The entire RT is not “unprotected” against chemicals, and inhaled substance can be removed mechanically by means of the bronchial mucociliary escalator even from lower compartments of the RT (Green et al. 1977). Below the bronchi, host defense by macrophages is becoming more important for local detoxification in the LRT.

Alterations within the lung/LRT may also be considered as local respiratory tract effects and can be accompanied by sensory irritation mediated via pulmonary vagal afferent C fibers. On the other hand, pulmonary edema, immunological effects (e.g., inflammation), or organ failure elicited from the disposition of xenobiotics in the LRT are severe and life-threatening events. As the mode of action of local effects in the LRT is different from that of sensory irritation in the URT, from which it can typically not be predicted, and since these effects do not manifest immediately (e.g., lung edema, inflammation by particles) unlike sensory effects that usually develop after a brief exposure to the chemical, this paper will exclusively focus on effects within the URT.

Thus, the next section will cover the anatomy, physiology, and sensory innervation of the eyes, the nasal cavity, and the larynx/pharynx region.

### Outer eye and ocular surface

**Fig. 2:**
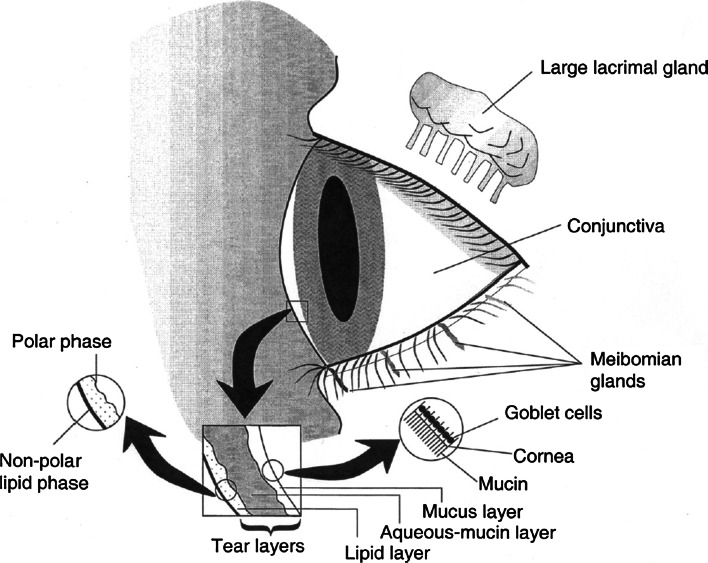
Various compartments of the outer eye that come in contact with volatile chemicals, and the composition of the tear film (cited from Wolkoff et al. 2003)

Various water-soluble chemicals (e.g., aldehydes) and also fine particles (e.g., sodium borate, calcium oxide) are known to cause eye irritation (Arts et al. 2006; Lang et al. 2008; Wolkoff and Nielsen 2010) by interacting with the ocular surface. Figure 2, taken from a review by Wolkoff et al. (2003), gives a macroscopic overview of the compartments of the eye that are in direct contact with the environment.

The central part of the cornea and the surrounding area of the sclera, both covered by the conjunctiva, constitute the ocular surface of the human eye. Both areas are composed of non-keratinized stratified squamous epithelium that is kept moist by different glands producing the tear film. The tear film consists of different layers and is formed during the eyeblink process. Its stability is crucial for the health of the eyes. Chronic dryness of the ocular surface resulting in inflammation is a cardinal symptom of Sjögren’s syndrome (Baudouin 2001), a systemic autoimmune disease affecting exocrine glands that produce tears and saliva.

Any thinning of the tear film destabilizes this protective layer, and its rupture shortens the breakup time (BUT) of the tear film. The BUT can be measured as the time interval from the last complete eyeblink until the occurrence of dry spots on the ocular surface. Usually, this BUT is within the time interval between two consecutive blinks (3–5 s) resulting in a normal blink frequency of 12–20 blinks/min (for more details see Wolkoff et al. 2003). Any reduction of the BUT, for instance by exposure to a chemical, needs to be counteracted by an “extra eyeblink.” Thus, the BUT is negatively correlated with the blink frequency. However, not the rupture of the tear film itself but the preceding sensation via the trigeminal nerve causes this blinking reflex. The ophthalmic (V1) branch of the trigeminal nerve innervates the conjunctiva and cornea of the eye, and the ocular surface is one of the most densely innervated structures of the body (Beuerman and Stern 2005; Veiga Moreira et al. 2007). Veiga Moreira et al. (2007) showed by means of retrograde tracing and electrophysiological techniques that both neurons from myelinated Aδ and unmyelinated C fibers receive input from the ocular surface. In accordance with intranasal trigeminal functions in humans, eye irritations are perceived as stinging and burning sensations (Hummel 2000). Such “eye symptoms” are often used in questionnaires assessing eye complaints related to environmental hazards (Franck and Skov 1991). However, prolonged stimulation of trigeminal receptors located on the ocular surface might cause neurogenic inflammation, a defense mechanism that is thought to be “the first line of defense for the ocular surface” (Beuerman and Stern 2005). The release of neuropeptides such as substance P or calcitonin gene-related peptide (CGRP) leads to a breakdown of the blood-tissue barrier facilitating the infiltration of immune cells like polymorphonuclear leukocytes into the tears (Beuerman and Stern 2005). Thus, overstimulation of the chemosensory system of the outer eye might be the starting point for inflammation and tissue damage. Such damage can be found in ocular surface diseases (OSNs, e.g., the hallmark in Sjögren’s syndrome) that are associated with loss of health-related quality of life and should be regarded as a severe health problem (Baudouin et al. 2008).

In regulatory toxicology, eye irritation after direct contact is an important end point for the classification of chemicals. Historically, the Draize test in rabbits has been used to investigate this aspect of acute toxicity. Even though the physiology of the human ocular surface is different from that of rabbits, eye irritation thresholds in humans and Draize test scores might be based on a similar mode of action (Abraham et al. 2003). However, the human eye is regarded as less sensitive than the rabbit eye (Barile 2010) so that the results of the eye irritation test in rabbits tend to overpredict the risk for humans. More recently, alternative methods have been developed and validated as screening tests for severe eye irritants and corrosive materials (e.g., bovine corneal opacity and permeability test, BCOP; isolated chicken eye test, ICE; Barile 2010) including in vitro models using chemical-sensing ion channels like the transient receptor potential vanilloid type 1 (TRPV1) receptor (Lilja and Forsby 2004; Lilja et al. 2007), but little experience has been gathered in routine testing of such methods so far. These tests give a measure of the severity of sensory irritation effects of chemicals on the eye, and Draize test scores have been shown to be in good agreement with human eye irritation thresholds (Abraham et al. 2003). Eye irritation thresholds and nasal pungency thresholds as a measure of sensory irritation were found to correspond closely to each other in humans (Cometto-Muñiz and Cain 1995). Therefore, the eye irritation potency is also regarded as predictive for the potency of irritation effects in the URT. There is not always a consistent correlation between Draize test scores and potency in sensory irritation tests, like the RD_50_ bioassay (Ciuchta and Dodd 1978), but empirically, substances classified as irritating to the eye based on the Draize rabbit eye test can reasonably be expected to act as local irritants in the URT, too. As alternatives to the Draize test, the BCOP test (OECD Test Guideline 437), the ICE test (OECD Test Guideline 438), and the Fluorescein Leakage test (TG 460) have found acceptance in regulatory toxicology. The acceptance of these tests is based on their capability to predict eye irritation with high severity and even corrosion, used to characterize classification category 1 of eye effects in globally harmonized system (GHS). Insofar, a positive test result in one of the alternative test systems can be expected to be predictive for a local irritation effect in the URT. However, there is no correlation between the scores obtained in these tests and the potency in the RD_50_ bioassay.

While the human ocular surface is a relatively homogenous tissue, the anatomy of the nose is far more complex. The following chapter provides only a brief overview and the reader is referred to the recently published volume “Toxicity of the nose and the upper airways” (Morris and Shusterman 2010) of the book series on target organ toxicity.

### The nasal cavity

The nose is the primary entry for inhaled air and is divided into two air passages separated by the nasal septum. While many rodent species are obligate nose breathers, humans can switch between nasal and oral breathing. The nasal-to-total ventilation rate is markedly reduced especially during exercise, and at moderate workload levels [in females at approximately 20 % of the maximal physical work capacity (PWC_max_); in males at approximately 40 % PWC_max_, corresponding to 22–23.5 l/min or 10 m^3^/8 h, the default ventilation rate assumed in the setting of OELs], the nasal contribution is reduced to 79 and 67 %, respectively (Bennett et al. 2003). Nevertheless, a great proportion of the total ventilation will pass the different epithelia of the nasal cavity even in situations with moderate workload.

The nasal passage extends from the nostrils to the nasopharynx and the nose functions as a “scrubbing tower” for the lower compartments of the RT. Due to physiological (e.g., cellular composition of the respiratory epithelium) and anatomical features (e.g., turbinates), the nose filters, warms, and humidifies the inhaled air and also effectively absorbs water-soluble volatile chemicals, traps inhaled particles, and metabolizes airborne xenobiotics. In general, two different epithelia can be found in the nasal cavity. The respiratory and olfactory epithelia cover different regions of the medial and lateral walls of the nasal cavity, and serve different functions, and thus, their cellular architecture and molecular functionality are also diverse.

#### The respiratory epithelium (RE)

The cellular composition of the epithelium gradually changes from the nasal valve to the posterior parts of the nasal vault. Squamous epithelium without microvilli covers the intranasal walls of the anterior part. The anterior tips of the turbinates provide the transition from the squamous to transitional and finally to pseudostratified columnar ciliated epithelium, lining the remaining parts of the nasal cavity except the roof, which is covered with the olfactory epithelium (OE).

The majority of cells composing the RE are of three types: basal cells, goblet cells, and columnar cells which are either ciliated or not. Basal cells have some ability to differentiate into other cells types, i.e., their function is associated with repair and regeneration of the RE after any kind of tissue damage. Goblet cells produce secretions that contribute to the mucous blanket. The major function of the ciliated cells is moving up the mucus to the pharynx, except in the region anterior to the inferior turbinate, where transport is anterior. The non-ciliated cells are thought to exhibit high metabolic activity, as they are rich in cytochrome P450 enzymes and esterases. Since most of the knowledge about the metabolizing capacity of the nose has been derived from rodent studies, some pronounced species differences have to be considered in the species-to-species extrapolation. In contrast to the human RE, the rat RE additionally contains brush cells supposed to have a neurological and sensory function. Furthermore, the goblet and the non-ciliated cells in the transitional epithelium contain abundant smooth endoplasmic reticulum (Harkema et al. 2006), suggesting that these cells may have metabolic capacities for certain xenobiotic-metabolizing enzymes (e.g., cytochrome P450 enzymes, carboxylesterases, aldehyde dehydrogenases, epoxide hydrolases, and glutathione S-transferases). Therefore, the metabolic capacity might be higher in the rat than in the human nose (for details, see Chapter 5 in Morris and Shusterman 2010).

Like the tear film covering the ocular surface, the mucus blanket covers the RE. It is composed of a lower, serous sol layer in which the cilia beat and a viscoelastic or gel layer is located above the cilia. The nasal mucous fluid is produced constantly by the various glands and is moved from the nasal cavity through the nasopharynx into the pharynx and then swallowed via the esophagus. The cilia beat in a fixed direction in a two-stroke pattern: an effector stroke, in which the cilia straighten, contact the gel layer, and moves the mucus; and a recovery stroke, in which the cilia bend and move in the watery sol layer.

This system is known as mucociliary clearance and serves important protective functions: It clears the nasal cavity of secretions and trapped particulates, provides water for humidification, and is known to exhibit a strong antioxidant activity. Furthermore, it is the first line of defense against bacterial and viral infection, as components of the mucus include IgA, IgG, IgE, histamine, albumin, lactoferrin, lysozyme, and cellular debris (Sahin-Yilmaz and Naclerio 2011). Mucociliary clearance rates of 3–25 mm/min have been shown in healthy humans (Mygind and Dahl 1998). Thus, normal mucociliary transit time takes between 12 and 15 min (Andersen and Proctor 1983). Transit times of more than 30 min are considered to be abnormal and are indicative of an impaired mucociliary clearance. In humans, mucociliary clearance can be assessed by the saccharine test (Muttray et al. 2002).

In the nose of rats, mucociliary clearance velocities of 1.1–5.9 mm/min were measured (Krinke 1999). Even though the speed of cilia-driven transportation is higher in humans than in rats, the transportation distance is longer, too.

The sites of certain toxicant-induced nasal injuries also differ between rats and humans because of intranasal regional differences in the amount of neutral and acidic mucosubstances. In humans, an increasing anterior-posterior gradient can be found with higher amounts in the distal nasopharynx area than in the anterior nasal airway. In contrast, the rat produces considerably more of these mucosubstances in the anterior septal region than in the nasopharynx area (Harkema et al. 2006).

#### Sensory innervation

The RE in the nasal cavity is innervated by the trigeminal nerve. Humans are aware of the stimulation of the free nerve endings/receptors by feeling sensations like burning, stinging, warmth, coolness, or itching (Hummel 2000). The signal transduction is predominantly mediated via multimodal ion channels (e.g., transient receptor potential ion channels like TRPV 1–4) that can be activated by temperature, irritants (chemicals), pH changes, or endogenous inflammatory mediators (Story 2006). These chemical-sensing receptors are not exclusively expressed in the trigeminal nerve. The whole somatosensory system of the peripheral nerves strongly depends on TRP channels. There are 28 TRP channels in mammals, detecting endogenous and environmental stimuli, such as temperature, mechanical forces, chemical stimuli, and pain (Vandewauw et al. 2013). These authors showed in their recent comparison of dorsal root ganglia (DRG) and trigeminal ganglia (TG) of mice that only the mRNA of TRPC1, TRPM4, TRPM8, and TRPV1 was differently expressed in the various ganglia.

Afferent fibers of the trigeminal nerve also supply the mucous membranes of the eyes, oral cavity, and nasopharynx. This chemical sense has been termed “chemo-somatosensation,” “chemesthesis,” or “common chemical sense.” Recently, Shusterman and Hummel (2009) proposed the more neutral terminology “trigeminal chemoreception” for all feelings associated with the stimulation of cranial nerve V (Shusterman and Hummel 2009). Trigeminal chemoreception is considered the most important sensory pathway in the context of *sensory irritation* (see Part B of the manuscript). Various reflexes (e.g., sneezing) and defense mechanism (e.g., neurogenic inflammation) are associated with the stimulation of free nerve ending of the trigeminal nerve in the nasal cavity.

#### The olfactory system

At the very top of the nasal cavity, beneath the cribriform plate of the ethmoid bone, a small area of the human nasal cavity (approximately 500 mm^2^) is covered by the OE. In humans, the OE occupies approximately 3 % of the nasal cavity, while in rats this tissue covers 50 % of the intranasal surface and extends also to more anterior parts of the nasal cavity. The OE is mainly composed of three epithelial cell types: the olfactory sensory neurons (OSNs), the supporting (sustentacular) cells, and the basal cells. The OE is one of the rare CNS structures where adult neurogenesis has been confirmed. OSNs undergo apoptosis and are able to regenerate due to the capacity of progenitor cells in the basal cell layer of the OE to proliferate and differentiate into mature OSNs. Thereby, the olfactory function is maintained even though inhaled xenobiotic agents may have induced severe cell injury and death of OSNs. Several studies have suggested that inflammatory signaling pathways may play a key role in the regulation of OSN regeneration. The baseline turnover rate has been proposed to lie between 28 and 30 days, yet more recent studies have shown that many OSNs are more long-lived (Harkema et al. 2006). In humans, this turnover process fails with age, and the surface area of the OE as well as the number of olfactory receptor neurons (ORNs) declines. Sustentacular cells contain abundant smooth endoplasmic reticulum and xenobiotic-metabolizing enzymes (e.g., cytochrome P450 enzymes, flavin-containing monooxygenases, *N*-acetyltransferases). They are supposed to contribute to the detoxification of inhaled xenobiotics and to play a role in the chemical interaction between odors and their olfactory receptors (ORs) by influencing the regulation of the ionic composition of the overlying mucous layer. Like the sustentacular cells, the Bowman’s glands contain many xenobiotic-metabolizing enzymes. Furthermore, they contain copious amounts of neutral and acidic mucosubstances and are solely responsible for the production and secretion of mucus covering the surface of the OE.

ORNs are predominantly located in the OE. The cilia of these neurons extend to the lumen and their cell membranes contain the ORs. Only one type of OR is expressed in each ORN, and the axonal projections of similar ORNs converge on glomeruli located in the olfactory bulb. Thus, the ORNs are neuronal structures that are in direct contact with all sorts of contaminants of the inhaled air (e.g., bacteria, viruses, volatile chemicals). Approximately 1,000 genes and pseudogenes encoding about 350 different and functional ORs are known in humans (Mombaerts 2001) and with this repertoire of odorant-specific receptors “we can detect hundreds of thousands, if not millions, of distinct odors” (Mombaerts 2001, p. 493). However, one has to bear in mind, that during normal breathing only 15 % (measured in rats; corresponding to 7 % in humans) of the inhaled air reach the OE (Kelly et al. 2000) via the dorsal medial airflow pathway, while a larger proportion just passes the RE via the lateral/ventral airflow pathway (Morris et al. 1993). Because of this, segmentation the OE of humans is more protected from exposure to chemicals than the respiratory epithelium. However, the sensitivity of the olfactory system is enormous, and for some chemicals such as skatole/3-methylindole, as few as 10 molecules might be sufficient to trigger an action potential at the receptor level and the subsequent detection of the odor by the central nervous system (Hatt 2000). This sensitivity is partly achieved by actively sniffing the odorants and increasing the airflow of the dorsal medial airflow pathway (Kareken et al. 2004; Sobel et al. 2000).

As pointed out previously, for the majority of volatile chemicals the olfactory system is more sensitive than trigeminal chemoreception (Shusterman 2001). This assumption has been confirmed for various irritants used in working environments as shown by comparing the respective odor and lateralization thresholds (van Thriel et al. 2006a).

### Larynx/pharynx

A mixture of afferent fibers from the glossopharyngeal nerve and the vagus nerve mainly innervate pharynx and larynx. Particularly, the superior part of the pharynx and parts of the oral cavity are solely innervated by the glossopharyngeal nerve. On the contrary, the inferior part of the larynx is exclusively innervated by the vagus nerve. Additional receptors are scattered in the airway walls within these compartments of the URT, and the surrounding muscles become more important. In the pharynx, a sniff-like aspiration reflex mediated by receptors in the squamous cell epithelium of this region protects lower compartments of the RT. Receptors in the larynx that respond to changes in osmolality are important in mediating swallowing. Nevertheless, multimodal receptors responding to chemical and mechanical stimuli (pain receptors), comparable to the intranasal receptors, also contribute to the manifold reflexes (e.g., by increasing mucus secretion or causing cough) that can be elicited by sensory nerves in these parts of the URT (Widdicombe 1982). The TRP channels (especially TRPV1) of this sensory pathway are mainly involved in coughing, a defense mechanism of the airways that is supposedly associated with sensory hyperreactivity (Millqvist 2000). From a physiological/biological point of view, these sensory-mediated airway reflexes, but also the intranasal reflexes like sneezing, nasal blockage (airflow obstruction), secretion (with or without associated inflammation), and mucociliary clearance, are important defense mechanisms protecting the lower parts of the RT, especially the alveoli, from harmful effects.

As in the case of the nose and other mucosal surfaces, the most rostral areas of the larynx lined by stratified squamous epithelial cells are those that are most directly exposed to inhaled substances. The thickness of the epithelium and the inherent resistance to damage of its squamous surface layers provide more protection than other types of epithelia. Nevertheless, the stratified squamous epithelium lining the rostral larynx of rodents is still more susceptible than that in other areas of the oropharyngeal cavity because it lacks keratin or is poorly keratinized under normal conditions compared to oral or nasal mucosa under normal conditions. Thus, exposure to irritants can induce edema, inflammation and, if prolonged and severe enough, blistering, necrosis, and epithelial sloughing. The epithelium lining the base of the epiglottis where the transition from stratified squamous epithelium to RE occurs in rodents is the area of the laryngeal epithelium that is most susceptible to damage from inhaled materials. The normal mucosal epithelium at the base of the rodent epiglottis consists of two to three layers of a mixture of ciliated and non-ciliated columnar cells, with no definite basal cell layer. A small area in the ventral midline at the rostral and caudal borders of the submucosal glands may be covered by squamous epithelium, but these areas do not have the prominent basal cell layer typical of stratified squamous epithelium (Renne and Gideon 2006).

### Biological relevance of the trigeminal and olfactory system

The *trigeminal system* is involved in pain sensation, and various nociceptors are expressed intranasally (Julius and Basbaum 2001). Pain is a useful warning system in all species and the sensation of pain triggers appropriate protective responses, mainly achieved via reflexes, as described previously. Thus, the response patterns are similar within and across species, resulting in a low inter- and intraspecies variability with respect to the biological significance of the sensory input. One approach to determine the activation of trigeminal chemoreceptors in the nasal cavity across species is to measure of the negative mucosa potential (NMP, Kobal 1985). The NMPs, elicited by 45 or 60 % of CO_2_, increased in rats by approximately 50 % (Thürauf et al. 1991). In humans, a comparable increase in CO_2_ resulted in an almost identical increase of the NMP (Hummel et al. 1998). Particularly, the higher CO_2_ concentration was perceived as painful (Hummel et al. 1998), revealing the biological relevance of trigeminal chemoreception. However, such perceptional aspects of trigeminal chemoreception are not assessed in inhalational exposure studies in rodents, but the NMPs, the electrophysiological correlates, can be used as comparable measures.

The functional role of the *olfactory system* in humans is less clear. Three categories of function related to ingestion, avoidance of environmental hazards, and social communication have recently been described (Stevenson 2010). Because of this broad functional spectrum, it is obvious that a large variety of differently concentrated odors have to be detected by the olfactory system. Moreover, there is no conclusive evidence, at least in humans, that the aforementioned categories of function are accomplished via reflexes of the olfactory system. Thus, learned response patterns (e.g., established via classical conditioning) might trigger “automated” responses (e.g., food avoidance) to certain odorants. Compared to the more uniform, trigeminal-mediated “pain” response, odors are capable to provoke a variety of behavior associated with the avoidance of environmental hazards (e.g., involuntary attention shift toward the odor/odor source). When sensory irritation effects are assessed for deriving exposure limits, the avoidance of excessive annoyance due to obnoxious odors might serve as basis for setting an OEL (e.g., DFG MAK list).

### Odor effects in humans

In animal studies, odor effects are usually not explored even though odor avoidance behavior is used as readout in neurobehavioral tests (Cloutier et al. 2006). Therefore, odor effects are usually investigated in human volunteer studies and subjective reports (e.g., ratings of odor intensity and quality) are the major source of information. Such information is subjected to various non-sensory factors (e.g., personality, pre-experiences with and/or information about the chemical), as shown repeatedly in chemosensory research (Dalton 1996, 1999, 2002, 2004). Thus, the use of such data in toxicological risk assessment is tricky. Nevertheless, odor annoyance should be defined in the context of this paper. Based on the general definition in the framework of quantitative risk assessment, a definition could read like odor annoyance is a feeling of displeasure associated with any agent or condition identifiable by the olfactory system that is believed by the person to affect adversely an individual or a group (Koelega 1987).

The critical aspect of this definition is the phrasing … believed to affect adversely … that pinpoints to the implicit shortcoming that there is no objective or physiological/biological marker of adverse odor effects. There is no reflex based on the stimulation of olfactory receptors, and thus, physiological readouts comparable to those of the three other cranial nerves innervating the RT are not available. In some recent papers (Kleinbeck et al. 2008; Hey et al. 2009; Rohlman et al. 2008), the use of neurobehavioral methods has been proposed to assess adverse effects of odors in the working environment. However, a general evaluation of this approach is still lacking.

### Species differences

#### Physiological factors

In human risk assessment, laboratory animal studies are the major source of evidence especially when evaluating health effects of chronic exposures. That is also true for local effects in the URT. In case of interspecies extrapolations, differences in anatomy, physiology, and breathing patterns have to be considered because they determine the dose delivered to the target site (Carey et al. 2007). Some interspecies differences with respect to anatomy and physiology have already been described in previous sections (e.g., size of the OE; distribution of squamous, transitional, and respiratory epithelium), but interspecies differences related to airflow dynamics have not been addressed so far. The distribution of airborne toxic agents within the nasal cavity is of special interest since different airflow velocities caused by anatomical features like the three turbinates largely affect both the deposition and the chemosensory perception of inhaled chemicals (Kelly et al. 2000). In humans, these structures are relatively simple in shape compared to the rodent nasal turbinates that have complex folding and branching patterns. In addition, the types and distribution of nasal surface epithelia differ among species (Harkema 1991). Models of human and rat nose have shown that the air stream over the human olfactory epithelia amounts to only 50 % of that of the rat (Frederick et al. 1998). This would be an argument in favor of a reduced variability factor for substances acting on the olfactory mucosa. Because of these anatomical characteristics of the rodent and the human nose and their effects on airflow and deposition, the extrapolation from rodents to humans should be based on fluid dynamics models to estimate the local concentrations of the compound (Schroeter et al. 2006; Kimbell et al. 1993). These models are available for several compounds (Corley et al. 2012; Schroeter et al. 2006; Sweeney et al. 2004), but due to physicochemical differences affecting the preference for a specific epithelium type (e.g., olfactory or respiratory; Garcia et al. 2009), general conclusions about species differences are difficult to draw.

#### Intranasal toxification and detoxification

As mentioned in one of the previous sections, nasal tissues have a capacity to metabolize airborne xenobiotics. Local biotransformation can thereby profoundly influence their toxicity. Compared to humans, the nasal tissue of rodents differs markedly with respect to enzymology. Particularly, rodents express high levels of cytochromes P450 (CYP) and also of other enzymes such as carboxylesterases, epoxide hydrolases, and acetaldehyde dehydrogenase (Ding and Dahl 2003). In humans, cytochromes P450 enzymes have been detected in nasal respiratory and olfactory mucosa (Ding and Kaminsky 2003), but their activities are apparently lower than those in the respective rodent tissues. For certain compounds, such as beta-lactones, esterases capacities may exert a detoxifying activity. In such cases, the sensitivity of humans toward the toxic activity of the parent compound might be higher. However, many esters and lactones are quite inert as parent compounds, whereas their cleavage by carboxylesterases may generate carboxylic acids as a sequel products at the intracellular level that lead to tissue irritation. In those cases, humans should be less sensitive than rats. Acrylic esters also show (as parent compounds) a considerable electrophilic reactivity toward proteins and may thus be more irritating than the esterase-mediated cleavage products. Methacrylates are much less reactive toward proteins and are normally not sensitizing. Hence, these compounds should be more similar to saturated esters than to acrylate esters in respect of species differences.

Methyl methacrylate is hydrolyzed to methacrylic acid by carboxylesterases. Inhibition of these enzymes reduced the severity of nasal lesions induced by this compound in rats, indicating that the toxic agent is methacrylic acid. Since the activity of carboxylesterases in humans is lower than that in rats, it can be assumed that tissue irritation occurs only at higher concentrations in man. *N*-butyl acetate is hydrolyzed to acetic acid and *n*-butyl alcohol. Because of the aforementioned species differences in enzyme activity, the metabolite acetic acid might cause tissue irritation at lower concentrations in rodents than in humans. The situation is different with acrylates, as the parent compound (e.g., ethyl acrylate, human NOAEC 2.5 ppm, see Part III) is more irritating than the cleavage product acetic acid (human NOAEC 10 ppm, Ernstgard et al. 2006). This is due to the highly reactive alpha, beta-unsaturated Michael system of the acrylates that is more irritating than the cleavage product, whereas the Michael system of methacrylates is known to be less reactive compared to that of acrylates.

Moreover, TRP channels in free nerve endings of the trigeminal system might only be activated by certain metabolites. Lanosa et al. (2010) showed that sensory irritation in mice induced by styrene was reduced after inhibition of CYP with metyrapone (measured by a RD_50_-like paradigm) and that this effect was mediated via a specific TRP channel as revealed in TRPA1 −/− knockout mice. The same results were obtained when naphthalene was used to induce sensory irritation.

Taken together, these results provide evidence that substances might be metabolized to reactive intermediates by CYP enzymes located in the URT. In these cases, species differences in CYP activity must be taken into account if respective data are available.

#### Deposition in nasal tissues

Acrylic acid has been shown to be deposited in the human nose to a lesser degree (52 %, Frederick et al. 1998) than in the nose of rats (97 %, Morris and Frederick 1995).

In contrast, according to calculations of Csanády and Filser (2007), the concentrations of propylene oxide are similar in the nasal mucosa of humans and rats when both species are exposed to the same concentration at rest, taking into account the metabolic detoxification of this compound in rat mucosa but not in human mucosa.

Selected differences in morphological and physiological features between rats and humans are summarized in Table 1. The proposed reference values vary depending on sex, body weight, and size.

**Table 1:** Differences in anatomy, physiology, and air flow dynamics between humans and rats

	Human	Rat
Morphology	Three turbinates in the nasal cavity with simple shape	Several turbinates with complex branching and folding patterns
	Average percentage of OE: 3 %	Average percentage of OE: 50 %
	Low metabolic capacity in the RE because of the lack of non-ciliated cells in the transitional epithelium	High metabolic capacity of cells located in the RE
Physiology	Nasal and oral breathing	Obligate nose breathers
	Pulmonary ventilation: 7.5 l/min (Arms and Travis 1988)	Pulmonary ventilation for 260 g rat: 0.2 l/min (Bide et al. 2000)
	Air flow over OE: 7 %	Air flow over OE: 15 %

For more details, see Morris and Shusterman (2010).

These species differences will be of relevance when recommendations for human risk assessment in the working environment will be given (see Part 3 of the overview). In general, the anatomy of the URT of rodents might be associated with a higher sensitivity for local effects in these species.

In summary, the first section of this overview summarized the physiology of the URT and the outer eye in relation to the possible target tissues of locally acting chemicals. It has been highlighted that the various compartments of the URT and the mucous membranes of the eyes are (a) richly innervated and equipped with different sensory receptors and (b) possess a heterogeneous mixture of different types of epithelia that are composed of various cell types serving diverse functions (e.g., local detoxification). Thus, sensory and non-sensory targets may be the starting point of a process that, under certain circumstances, leads to adverse health effects both in animals and humans. In the next section, a conceptual model of these processes will be proposed and definitions of the key terminology will be provided.

## Part 2: Local effects of irritants on the upper respiratory tract and the mucous membranes of the eyes in the working environment—sensory and non-sensory origins

### A model for two modes of action

**Fig. 3:**
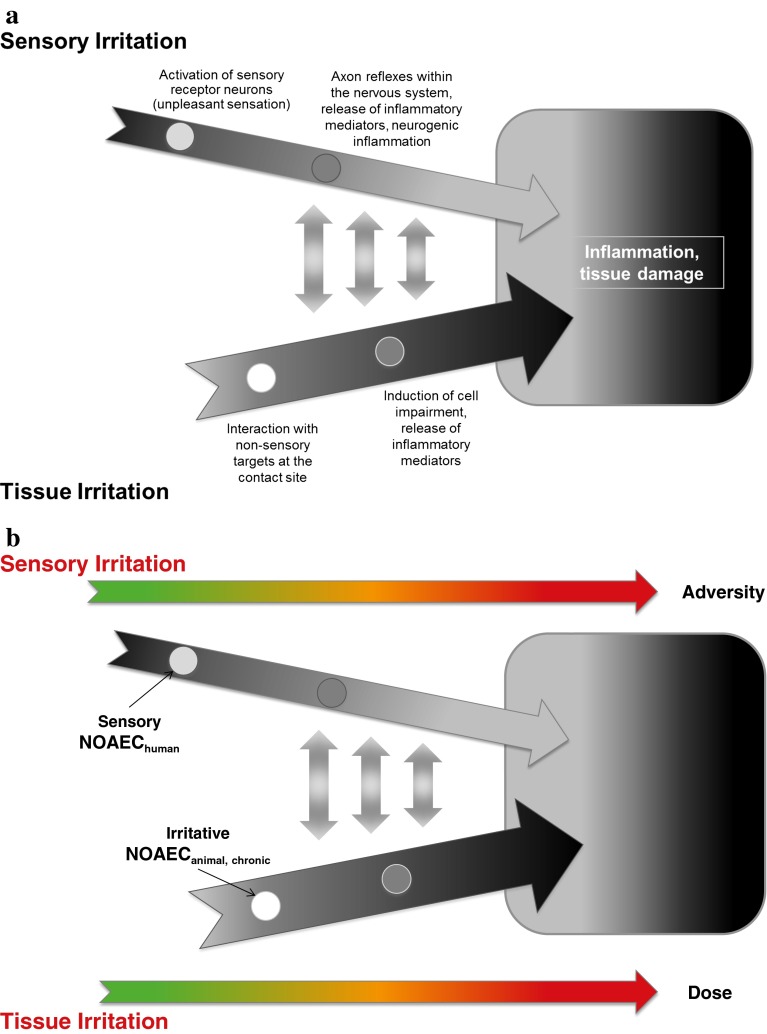
**a** A scheme of two interlinked pathways relevant for the causation of local effects in the upper respiratory tract and the mucous membranes of the outer eye. **b** Localization of two different NOAECs on the two-pathway model supposed to underlie local effects on the respiratory tract and the mucous membranes of the eyes

Effective host defense against irritation of the URT is based on a time-shared interaction of the peripheral nervous and the innate immune system. Both systems possess receptors for the detection of exogenous and endogenous hazards (e.g., xenobiotics, pathogens, hypoxic environment), and especially in the peripheral nervous system, some of these receptors are co-localized on afferent fibers (Chiu et al. 2012). In Fig. 3a, two pathways of local effects of irritants and their mutual interaction are schematically illustrated.

The first pathway (sensory irritation in Fig. 3a) is a receptor-based interaction of the chemical with sensory neurons located in the respiratory and olfactory epithelia and the cornea/conjunctiva. Thus, the targets are neuronal cells and their ramifications in the mucous membranes. Consequently sensory irritation is a very rapid process that can proceed within milliseconds from stimulation to awareness. However, at concentrations exceeding a certain effect threshold, the stimulation of neuronal receptors is supposed to be also the initial step of the first mode of action leading to adverse health effects on the URT and the eyes. In regulatory toxicology, this pathway is usually linked to the term *sensory irritation* (Shusterman and Hummel 2009) caused by the repeated or strong stimulation of the chemosensory systems as described in Part 1 of this paper. Regarding dose/concentration dependency in men, but also in laboratory animals, *sensory irritation* is usually caused by lower concentrations, while higher concentrations and prolonged high exposure (see Part 3) might produce *tissue damage* in the URT (with the exception of compounds with a weak or without a sensory warning effect; see second pathway). At low concentrations, acute effects such as olfactory (annoyance) or trigeminal (stinging, itching) perceptions can be considered as *unpleasant sensations*, which are, however, totally reversible. In Fig. 3a, the adversity of the sensory-mediated effect increases from left to right, and the initial unpleasant sensation might become an adverse health effect due to the cascade of physiological defense mechanisms and finally tissue damage. However, physiological events in the first pathway can be detected at very low concentrations. This fact is visualized by an earlier start of the arrow. Furthermore, sensory irritation is a very fast reaction. A thin arrow visualizes this speed component.

The second pathway (tissue irritation in Fig. 3a) starts with interactions of airborne chemicals (e.g., dibutyl phthalate, DBP) and non-sensory targets (e.g., epithelial cells). In contrast to the first pathway, these targets are not exclusively located on neuronal cells, and thus, all cell types composing the mucous membranes of the URT and the eyes can be affected. Therefore, this pathway is linked to the term *tissue irritation*. “Induction of cell impairment” can include damage of cell membranes and cytoskeleton, impaired energy charge or disturbances of the cell homoeostasis. It is unclear which of these effects governs inflammatory cell signaling. Compared to the first pathway, the irritation process is slower, lasts minutes or rather hours or days, and usually starts at higher concentrations or with prolonged or repeated exposure. This is visualized in Fig. 3a by a thicker arrow and a later start. Again, the adversity of the effects increases along this pathway.

Moreover, the two proposed response cascades are interlinked (indicated by the double-headed arrows): Effects along the tissue irritation pathways can be sensed by nociceptors (e.g., TRPV1) that are also involved in the first pathway. In parallel, effects along the sensory irritation pathway (e.g., inflammation) might encroach on non-sensory epithelial cells. At a certain point in the proposed model, these overlaps hamper the distinction between the starting points of the local effect.

While the short-term stimulation of the sensory irritation pathway is thought to be totally reversible (Hummel 2000), it is considered the human lowest observable effect concentration (LOEC, based on physiological measures of nerve stimulation). Because adverse health effects do not occur at this concentration, the human sensory LOEC can be used as surrogate for the no observed adverse effect concentration (NOAEC). Repeated exposures to concentrations above this NOAEC resulting in prolonged nerve stimulation (within hours) that might occur in working environments can trigger a response cascade leading to chronic adverse health effects.

Neurogenic inflammation plays an important role along the sensory irritation pathway (Beuerman and Stern 2005; Lacroix and Landis 2008). It reflects the transition from pure sensory, reversible effects (including sensory-mediated reflexes like coughing, sneezing, and lacrimation) to more general and inflammatory defense mechanisms against the toxic action of xenobiotics, likewise seen along the tissue irritation pathway. A recent review describes the similarity between the molecular recognition pathways of nociceptor neurons with those of immune cells and the direct communication between these cell types in response to danger (Chiu et al. 2012). As an immediate first responder (e.g., feeling the pain), the nervous system is equipped with neuronally released mediators (e.g., substance P) signaling to vascular endothelial cells to increase blood flow and vascular leakage. Moreover, sensory nerves are equipped with cytokine receptors (e.g., IL-1β or TNF-α receptors) that increase membrane excitability by intracellular MAP kinases signaling. Thus, a non-sensory pathway, namely the release of cytokines, might facilitate an increase of sensory perception. In humans, these subsequent effects of sensory stimulation might be considered the lowest observed adverse effect concentration (LOAEC).

In parallel, the second pathway might also start as a reversible, adaptive response that leads to a chronic adverse health effect when the physiological repair capacities become overwhelmed. Because of these capacity-depleting effects even at the initial steps of the second pathway, the exposure duration might be more important than in the first pathway where a more threshold-like mechanism indicates the switch from sensory to non-sensory responses/reflexes.

Regardless of the initial steps, the tissue irritation and the sensory irritation pathways might cause chronic effects like inflammation and tissue damage mediated by the innate and adaptive immune system (Tan et al. 2010). Both pathways can become indistinguishable at this final point. This is visualized in Fig. 3a by convergent arrows that end in the same box displaying the final step of both pathways.

Based on this conceptual model and in order to conceptualize the theoretical framework of local effects on the RE and the eyes, the following definitions of the key terms are proposed:

*Sensory irritation* is defined as sensory-mediated responses of nervous system pathways in the target site, in humans accompanied by trigeminal and olfactory chemoreception (feeling of the exposure) that can trigger a broad array of defense mechanisms protecting the RT and the eye. These primary reactions are thought to lead only to reversible alterations of the physiology/biology of the target site. However, prolonged exposure at a concentration exceeding a certain effect threshold (e.g., of disturbing homeostasis or overpowering metabolic detoxification) might deplete the physiological resources for the reflex-like defense processes, and additional responses, mainly from the immune system, might become important.

*Tissue irritation* is defined as (a) damage of proteins, membranes, or organelles of the non-sensory cells in the respective epithelia, leading to (b) macroscopically or microscopically visible changes (e.g., dry spots, redness) at the outermost epithelial layers of the target site, e.g., the mucosa of the URT or the mucosal membrane of the eyes. While minimal cell damage can be repaired by removal of damaged organelles via cellular stress response pathways or autophagy, more severe damage is associated with structural changes (e.g., swelling of the cell membrane). These changes can deteriorate further, resulting in necrosis or in the more controlled process of apoptosis. More severe levels of irritation might also be accompanied by inflammation.

Like in many other areas of toxicology, Fig. 3a also displays the difference between acute and chronic effects (left to the right side of Fig. 3a) of volatile chemicals causing local effects on the mucous membranes of the URT and the eyes. However, information about sensory irritation is often lacking within the context of toxicological risk assessment or is assessed in humans, e.g., in experimental exposure studies with human volunteers. Such studies are usually designed to derive a sensory NOAEC (initial starting point of the first pathway) or LOAEC and short-term exposures (2–4 h) to concentrations known to stimulate the chemosensory systems are investigated. In contrast, animal experiments can address adverse irritant or inflammatory effects following acute, subacute, or chronic exposure toward high, cytotoxic concentrations. The effect assessment is usually based on histological and pathological effects. Pure sensory effects can also be assessed in laboratory animals (e.g., Morris 2002), but such studies are very rare.

In conclusion, the proposed model assumes that (a) sensory irritation occurs at lower concentrations than tissue irritation, (b) sustained exposure or high concentrations trigger a pathway-dependent response cascade, (c) certain steps lead to irreversible effects on both pathways, and (d) the two pathways become indistinguishable when morphologically and biochemically ascertainable changes occur.

Based on experimental inhalation studies in humans or animals, and sometimes even on epidemiological studies, risk assessors might be able to derive two kinds of NOAECs (Fig. 3b). Since human inhalation studies usually investigate effects associated with sensory irritation, LOECs and NOECs for the first pathway can be derived by statistical comparisons. After the evaluation of these studies by risk assessors, a sensory NOAEC_human_ can be derived (see upper part of Fig. 3b). Inhalation exposure studies in rodents may provide tissue irritation LOAECs and NOAECs (second pathway) that can be used to establish an irritative NOAEC_animal_ during the process of deriving OELs (see lower part of Fig. 3b).

#### Definition of the NOAECs

*Sensory NOAEC*_*human*_ is based on NOECs showing no statistical significant difference from a control condition in experimental, short-term exposure studies conducted with human volunteers. Such studies should investigate sensory irritation by using appropriate methods (see Doty et al. 2004). Guidance for the evaluation of such chemosensory effects has been given in the proceeding of the “adversity workshop” held in Cologne in 2005 (for editorial, see van Thriel et al. 2006a). Accordingly, a sensory NOAEC_human_ should be derived from reactions caused by trigeminal chemoreception and measured distortion free with physiological measures, e.g., eyeblink frequency or biochemical analysis of nasal lavage fluid. These physiological responses are based on sensory-mediated defense mechanisms/reflexes and are not adverse end points per se. However, if these defense mechanisms/reflexes are elicited continuously under high and prolonged exposure, they can result in adverse health effects. In combination with psychometric ratings of subjectively perceived symptoms, intensity estimates of irritation or odor perception give valuable input to improve the decision-making process for determining OELs. If only psychometric ratings are available, not only the statistical difference should be considered but also the magnitude of the effect. Since processes of sensory adaptation or temporal summation usually take place during a period of approximately 2 h (e.g., Cain et al. 1986; van Thriel et al. 2005), the exposure in such sensory NOAEC_human_ studies should continue for at least 2 h. Shorter studies can be used if it has been demonstrated that the effect reaches its steady state at an earlier time point. Since trigeminal chemoreception is subjected to temporal integration/summation (Wise et al. 2005, 2006), the time course of psychometric ratings across the exposure period might be used to discriminate between odor effects (e.g., reported annoyance) and sensory irritation (e.g., ratings of eye irritation). The studies should be conducted according to principles in experimental research (i.e., control for confounders, sequence effects etc.), and the sample size should be sufficient to detect even medium and sometimes small effects (e.g., by using techniques of statistical power analysis; Faul et al. 2007). As a rule of thumb, experiments investigating the response of volunteers to three or more exposure conditions are more sensitive. In this case, smaller sample sizes might be sufficient (e.g., group size approximately ≥12 volunteers). Epidemiological studies conducted in the working environment might also provide a sensory NOAEC_human_ if a clear association between measurements of exposure and sensory irritation exists.

According to this definition of the NOEC, the LOEC would be the first experimental condition (i.e., investigated concentration) that statistically differs from the control condition. Considering the magnitude of the observed effect at this LOEC and the biological mechanism underlying sensory irritations, regulatory agencies might use either real NOECs or LOECs associated with weak effects to derive a “sensory NOAEC_human_.”

The irritative NOAEC_animal_ is based on a statistically significant NOAEC found in chronic inhalation studies with rodents (2 years) by using biochemical and morphological examination techniques to identify even mild forms of tissue irritation/damage in the URT.

The evaluation of the proposed model suffers from a substantial lack of compounds for which sufficient human and animal data are available to establish an irritative NOAEC_animal_ and a sensory NOAEC_human_. In the following section, selected compounds will be presented that might be feasible candidates for such an empirical underpinning of the proposed model. It will be checked whether adequate human and animal data for indicating the two starting points of the model has been gathered for these compounds, and if so, whether rough estimates of the ratios between the two “effect thresholds” can be given. Previous assumptions underlying the existing default factors used in human risk assessment will be challenged.

## Part 3: Recommendation for the derivation of occupational exposure limits based on sensory irritation on the URT and the mucous membranes of the eyes

### General remarks

This part deals with the possibility to derive health-based occupational limit values for chemicals for which the most sensitive effect is sensory irritation of the URT or the eyes. As such, these effects always start as acute irritation at the exposed site (eye, nose, and throat) but can—when exceeding a certain concentration and exposure duration—also lead to cytotoxic and inflammatory effects.

It is noteworthy that if human data are available it is not used without considering animal data to form the full picture of the mode of actions and without taking into account the relevant effect thresholds seen in the animal data. The OEL setting process always requires a full review of all available toxicity databases on the substance in question. In a “case-by-case” approach, all relevant human, animal, and other experimental information as well as background data are assembled, and it is established which adverse effect(s) is(are) considered to be crucial for the setting of an OEL.

A substance can be considered to be a sensory irritant according to

- empirical results in humans (e.g., workplace exposure)
- physicochemical properties (e.g., pH value, reactivity) and structure–activity relationships
- animal inhalation studies
- animal irritation tests (eye, skin)

So far, only in vivo studies in animals and repeated exposures allow deriving a NOAEC for morphologically effects observable in the RT (irritative NOAEC_animal_). The results, however, do not exclude a sensory effect at lower concentrations. Sensory irritation of the eyes that might very well be more sensitive than the URT cannot be reliably observed in animal inhalation studies, and the magnitude of irritation seen in eye irritation tests (e.g., Draize test) cannot be quantitatively extrapolated to humans. In controlled human studies, usually no histological examination of the URT is possible but subjective and objective measures of sensory-mediated reflexes of the URT as well as eye irritation can be obtained (see Doty et al. 2004). Thus, animal and human studies complement each other on the effect continuum.

OELs can be set if

- Results from human exposure (controlled studies or workplace exposure) are available that allow a quantitative assessment. Ideally, human data have been obtained from experimental studies with controlled exposures and well-assessed end points of sensory-mediated defense mechanisms/reflexes such as eyeblink frequency. Though such studies are rarely available, they are an important basis for OEL setting, especially in those cases where the sensory end point is more sensitive than the morphologic end point. Human data can also be obtained from occupationally exposed persons. Such data may indeed be used for the derivation of OELs provided that at least some appropriate exposure measurements do exist. If good complete data from human exposure that allow the derivation of a NOAEC or LOAEC are not available, OELs should be evaluated on the basis of animal experiments. Human studies without controlled exposure such as workplace studies typically can help to verify the animal data.
- Results from animal inhalation studies with repeated exposure are available which evaluated adequately local irritant effects. Critical effects of an irritant are usually assessed based on histological signs of inflammation or other end points, e.g., lesions of the respiratory or OE, basal cell atrophy, hypertrophy of Bowman’s glands, or larynx metaplasia. In some cases, additional studies—in different species—may specifically address irritative end points such as nasal secretion or mucociliary clearance.

#### Inter-/intraspecies extrapolation

In most cases, OELs are derived from NOAECs/LOAECs from animal studies with extrapolation factors (EF) to allow for inter- and intraspecies variability. Ideally, results from chronic animal studies are available; in other cases, time extrapolation may be necessary.

Currently, for substances exerting systemic and local effects, the same EF, namely 1/5 for inter-/intraspecies (combined value) variability is used in the frame of the OEL and DNEL concepts in case of inhalation toxicity data as a starting point. This factor was proposed to cover the variability of toxicokinetic and toxicodynamic aspects between humans and also potential additional interspecies variability. It is, however, only based on limited evidence. ECETOC proposed an interspecies factor of 1 and an intraspecies factor of 1/3 for local effects (ECETOC 2003, 2010). In case that the OE is the target tissue within the URT, an interspecies extrapolation factor of 2 can be considered on a case-by-case basis. This consideration is based on the twofold higher airflow along the rat OE resulting in a twofold high tissue burden in rats as compared to human OE.

In the following, the magnitude of the extrapolation factor used for inter-/intraspecies variability (iEF) is evaluated by using examples of chemicals for which animal and human data on local effects could be obtained from the literature. Instead of trying to determine the variability between species and between human subjects, another approach was taken and the chronic N(O)AEC of the animal study for histology of the URT was compared with the human NOAEC for sensory irritation. An iEF was deduced from this comparison.

In the case of the human NOAEC, it has to be borne in mind that this concentration is not a no-effect-concentration, i.e., the subjects may report sensory effects at this concentration but the magnitude of the response is judged to be not adversely affecting the person. Even if the increase in the response compared to the control exposure is statistically significant, the magnitude of the increase is too low to be judged as adverse.

#### Exposure duration: time extrapolation

The European REACH framework has introduced so-called DNELs (derived no effect levels). For derivation, default factors to be used when no chronic studies are available are

- 1:6 for extrapolation from a subacute NOAEC,
- 1:2 for extrapolation from a subchronic NOAEC.

In case of similar NOAECs observed in subacute and subchronic studies, the default factor for extrapolation from subchronic studies is not applied, because in that case the concentration plays a more important role than the duration for the development of adverse effects.

These factors are based on the literature evaluations and represent the geometric means of ratios of NOAECs for subacute, subchronic, and chronic animal studies. ECETOC (2003, 2010) has questioned the magnitude of these factors and proposed not using a tEF for locally acting substances, because there are some examples where no lowering of the NOAEC with increasing exposure time can be seen in animal experiments (notably formaldehyde).

In contrast, the human sensory NOAECs/LOAECs are based on the stimulation of the chemosensory systems (see Part 1), often as acute inhalation study and they are supposed to provide estimates of reversible effects located at very early stages of the first pathway of the model (feeling of the exposure). Such early and pure sensory responses do not appear to be associated with any cross talk between the nervous and the immune system (Chiu et al. 2012) leading to non-sensory responses (e.g., release of cytokines). Based on some empirical evidence provided by experimental and epidemiological studies, initial indicators of sensory irritation do not add up over time, at least not in subeffective concentrations. Thus, additional time extrapolation might not be required if OELs are derived from human sensory NOAECs.

This assumption can be verified by comparing NOAECs of acute human exposures with exposures at the workplace, although the number of compounds that allow for such a comparison is limited.

For methyl methacrylate, a NOAEC of 40 ppm has been deduced from workplace data. The LOAEC has been reported to be >100 ppm (Röhm 1994 in DFG 2006). The acute human experimental NOAEC is 50 ppm (Table 2). Thus, in humans, acute and chronic NOAEC are the same.

**Table 2:** Results of the comparison for three substances with chronic inhalation studies in laboratory animals and human data from experimental studies

Substances	LOAEC (ppm) NOAEC (ppm)^a^ for histology of URT in animals			Human LO(A)EC (ppm) and NO(A)EC (ppm)^a^	Chronic NO(A)EC/human NO(A)EC
	SA: subacute	SC: subchronic	C: chronic		
Ethyl acrylate	–	25	25	5	2 (5/2.5)
		–	**5**	**2**.**5**	
Formaldehyde	6	3	2	0.5	3.3 (1/0.3)
	**2**	**2**	**1**	**0**.**3**	
Methyl methacrylate	110	–	100	>100	0.6 (25/40)
	–		**25**	**40**	

^a^According to the definition of sensory NOAEC_human_ and irritative NOAEC_animal_. For some compounds, new data might be available that was not consider for OEL setting procedures yet (see “Substance” sections)

For calcium oxide, workplace data have shown a NOAEC at an average concentration of 1.2 mg/m^3^ (0.4–5.8 mg/m^3^) (Torén et al. 1996) which is in agreement with the experimental acute human NOAEC of 1 mg/m^3^ (Cain et al. 2004, 2008).

For disodiumtetraborate, workplace data (Hu et al. 1992; Wegman et al. 1994) show that exposure concentrations from 1.76 to 7 mg/m^3^ have a probability of 1 % to result in moderate or stronger nasal irritation (most sensitive target site). For the next higher exposure group 8.8–15.8 mg/m^3^, the probability was 8 %. The concentration range up to 7 mg/m^3^ can therefore be interpreted as a NOAEC. The human experimental acute NOAEC is 5 mg/m^3^ (Cain et al. 2004, 2008). Thus, acute and chronic NOAEC are quite similar.

From this albeit limited information, it can be deduced that the human acute experimental NOAEC is similar to NOAECs derived from exposures at the workplace.

### Procedure of evaluation

#### Substance selection

Substances were included which are under discussion in the MAK commission or in the UA III of the AGS due to unresolved questions regarding inter- and intraspecies variability and time extrapolation. As a result of this discussion, which included a literature search, 19 substances were identified known to be human irritants for which both human and animal data are available. However, these substances differed with respect to amount and quality of the database.

The available data were reviewed considering the following questions:

1. Is the sensory irritation effect the most sensitive end point?
2. Are data about morphologically and biochemically ascertainable changes from animal studies available and is the study quality sufficient?
  Which NOAEC and LOAEC were found for subacute, subchronic, and chronic studies?
3. Are data about sensory irritation from animal studies available?

The evaluation started with those three substances for which a good complete database—human as well as animal—is available: for ethyl acrylate, formaldehyde, and methyl methacrylate, an appropriate chronic animal study and a controlled human exposure study of at least 2 h are available and allow the quantitative assessment of sensory effects. The consideration of only substances with chronic animal studies in these cases avoids the necessity of time extrapolation. The ratio between LO(A)ECs and N(O)AECs of the animal and human studies was calculated to deduce an overall iEF (Table 2). It should be pointed out that the concentration spacing used in the studies influences this ratio markedly.

For the following substances, the NOAECs and LOAECs of the studies cited were taken from the respective documentation(s) of MAK values, except for studies which are included in the list of references.

*Ethyl acrylate* From a chronic inhalation study (6 h/day, 5 days/week, up to 27 months), a NOAEC of 5 ppm and a LOAEC of 25 ppm were derived based on non-neoplastic changes at the OE, hyperplasia, and inflammation of the Bowman’s glands in rats and mice (Miller et al. 1985). The alterations in the nasal mucosa were present in subgroups of rats and mice at the first interim evaluation after 3 months of exposure for rats and after 6 months for mice. There were no exposure-related changes in the nasal mucosa of animals exposed to 5 ppm. The results of an experimental human study showed significantly increased eyeblink frequencies (about 30 %) at peak concentrations of 10 ppm (0–10 ppm, i.e., 5 ppm time-weighted average exposure concentrations (*C*_TWA_). Analysis of nasal lavage gave a slight but not significant indication of neurogenic inflammation. After constant exposure to 5 ppm, substance P was increased threefold, and changes in eyeblink frequency were observed as well; therefore, 5 ppm were considered as LOAEC, and after varying exposure to 2.5 ppm *C*_TWA_ (0.5–5 ppm), a weak increase of 1.5 times was found, but not considered relevant. Exposure to 2.5 ppm and above led to severe odor annoyance (Blaszkewicz et al. 2010).

The ratio of the chronic animal NOAEC and the acute human NOAEC gives a factor of 2.

*Formaldehyde* Formaldehyde has well been studied in animals and humans. Investigations on irritation effects in mice and rats show that mice (RD50: 3–5 ppm) are more sensitive compared to rats (RD50: 10–30 ppm). From chronic inhalation studies in rats, NOAECs of 1 ppm (Woutersen et al. 1989) and 2 ppm (Monticello et al. 1996) could be derived. In a chronic inhalation study in mice and rats, effects were observed at all exposure levels (0, 2.0, 5.6, and 14.3 ppm, 6 h/day, 5 days/week, 24 months): rhinitis, epithelial dysplasia, and squamous metaplasia (Kerns et al. 1983). Therefore, here a LOAEC of 2 ppm is taken into consideration. The true NAEC is expected to be between 1 and 2 ppm. For this evaluation, a NOAEC of 1 ppm is taken into account. In subchronic studies, a NOAEC of 2 ppm can be derived from the study from Wilmer et al. (1989). The studies from Rusch et al. (1983) and Zwart et al. (1988) have found epithelial hyperplasia and metaplasia occurring already at a concentration of 3 ppm, the LOAEC for effects from subchronic studies. NOAEC and LOAEC for subacute exposures can be taken from the study of Monticello et al. (1991) who found epithelial hyperplasia and metaplasia at a concentration of 6 ppm, while at 2 ppm no effects could be observed. The human NOAECs are between 0.3 ppm (with peaks of 0.6 ppm) (Paustenbach et al. 1997; Lang et al. 2008) and 0.7 ppm (Müller et al. 2013). Since there are some indications for slight irritative effects at concentrations of 0.5–0.6 ppm, 0.3 ppm is considered as NOAEC and 0.5 ppm as LOAEC. The NOAEC_animal_ and the NOAEC_human_ ratio results in a factor of 3.3.

As mentioned earlier, new data are available for this compound but have not been evaluated for setting OELs.

*Methyl methacrylate* An animal NOAEC from a chronic inhalation study in rats (6 h/day, 5 days/week, 24 months) was set at 25 ppm. Concentrations at and above 100 ppm caused degeneration, atrophy, hyperplasia, and metaplasia in the OE, and hyperplasia and inflammation in the respiratory epithelium (EU 2002; Lomax et al. 1997). In a study with subacute exposures (Hext et al. 2001) from 1 to 28 days, toxicity at the OE was observed at concentrations of 110 and 400 ppm. However, effects after exposure to 110 ppm were fully reversible already during the exposure period. Since there is no clear explanation for this phenomenon, 110 ppm cannot be regarded as a distinct NOAEC.

A study in exposed workers, which involved determination of sensory irritation symptoms and rhinoscopy, revealed a NOAEC of 40 ppm (Röhm 1994 in DFG 2006). Symptoms of irritation were reported at concentrations above 100 ppm. This is in agreement with recent experimental human studies that found only weak irritation effects and moderate odor effects at concentrations up to 50 ppm (*C*_TWA_) (Muttray et al. 2007; van Thriel et al. 2010). These effects were limited to reports of irritation since none of the applied physiological measures (e.g., eyeblink frequency) showed a significant increase at this concentration.

The ratio of the chronic animal NOAEC and the acute human NOAEC is 0.6.

This rather low ratio of animal NOAEC/human NOAEC might be due to the lower activity of carboxylesterases in human nasal tissues that would suggest that humans receive a lower dose of the cleavage product methacrylic acid, which is responsible for the irritation effects (see Part 1). Additionally, the dose spacing in the animal study may have confounded the ratio.

In conclusion, the ratios between the tissue irritation NOAEC_animal_ and the sensory irritation NOAEC_human_ are given in Table 2 range from 0.6 to 3.3 and accordingly, a default interspecies extrapolation factor (iEF) of “3” is proposed.

In the following section, the validity of this iEF of 3 will be evaluated for compounds with good but partly incomplete data, i.e., such compounds for which an appropriate subacute or subchronic but no chronic animal study are available. Our calculations are based on the NOAECs from the human data (sensory NOAEC_human_) shown in Table 3 which are then multiplied by the iEF of 3. For compounds with no chronic animal study, it is necessary to extrapolate the chronic NOAEC. Applying the default factors for time extrapolation (tEF), theoretical values for the NOAECs for subchronic and subacute animal studies are calculated. The resulting product corresponds to the theoretical NOAEC for a chronic animal study. The resulting theoretical NOAECs for subchronic or subacute animal studies are compared with the real data derived from toxicological studies.

**Table 3:** Application of an iEF of 3 to a data set with good quality but without chronic exposure study in animals

Substances	Human LO(A)EC (ppm) and NO(A)EC (ppm)^a^	Application of interspecies factor (iEF) 3 to human NO(A)EC (ppm)	Application of time extrapolation factor (tEF)	LOAEC (ppm) NOAEC (ppm)^a^ for histology of URT in animals	
				SA: subacute	SC: subchronic
Acetaldehyde	–	50 × 3 = 150	tEF: 1 (SC → C)^b^	243	150
			150 × 1 = 150	**150**	50
	**50**				
Ammonia	–	25 × 3 = 75	tEF: 2 (SC → C)	–	250
	**25**		75 × 2 = 150	–	**200**
*n*-Butyl acetate	–	147 × 3 = 441	tEF: 2 (SC → C)	–	1,500
	**147**		441 × 2 = 882	–	**500**
2-Ethylhexanol	20	15 × 3 = 45	tEF: 2 (SC → C)^c^	–	–
	**15**		45 × 2 = 90		
				**120**	**120**
Hydrogen sulfide	–	5 × 3 = 15	tEF: 2 (SC → C)	80	30
	**5**		15 × 2 = 30	**30** (**3** h/day) = **10** (**8** h/day)	**10**

*SA* subacute, *SC* subchronic, *C* chronic

^a^According to the definition of sensory NOAEC_human_ and irritative NOAEC_animal_. For some compounds, new data might be available that were not consider for OEL setting procedures yet (see “Substance” sections)

^b^With tEF = 1 because in the subchronic study, effects were already seen after 4 days of exposure to 150 ppm

^c^With tEF = 2, although subacute and subchronic study yielded the same NOAEC, however, there was no LOAEC obtained in either study

Before getting to final conclusions about the applicability of the iEF, the five substances fitting our definition of a substance with good but partly incomplete data are evaluated.

*Acetaldehyde* A human NOAEC of 50 ppm was provided by a controlled acute study that did neither find self-reported irritation nor measurable inflammatory effects. The following parameter were investigated: mucociliary transport time before and after exposure, interleukin-1β and interleukin 8 in nasal secretions, as well as mRNA-levels of interleukins-1β, 6 and 8, tumor necrosis factor-*α,* granulocyte-macrophage colony-stimulating factor, monocyte chemotactic protein 1, and cyclooxygenases 1 and 2 in nasal epithelial cells were measured after exposure (Muttray et al. 2009).

A subchronic toxicity study in rats (Dorman et al. 2008) with interim examinations showed that effects were observed already at day 4; therefore, there is no need to include time extrapolation in the calculation.

 Interspecies extrapolation for sensory irritation effects with the iEF of 3 [as the effects were seen in the OE, a reduced iEF would be more appropriate to account for the lower burden in human OE (see also Teeguarden et al. 2008) and time extrapolation of 1] leads to an estimate of a subacute or subchronic NOAEC of 150 ppm. This is comparable with the NOAEC of 150 ppm found in a subacute repeated dose-study (6 h/day, 5 days/week, 4 weeks) in rats, based on histopathological findings in the OE, in particular loss of microvilli, thinning and disarrangement (Appelman et al. 1986), whereas 150 ppm was a LOAEC in a subchronic study (6 h/day, 5 days/week, 13 weeks) in rats based on minimal olfactory degeneration at 150 ppm (NOAEC 50 ppm). Mild inflammation and hyperplasia of the respiratory epithelium started at 500 ppm (Dorman et al. 2008). Based on the concentration of acetaldehyde in the OE and taking into account ALDH2 polymorphisms in humans, the NOAEC of 50 ppm found in the subchronic study with rats corresponds to a human equivalent concentration of 67 ppm (Teeguarden et al. 2008). This calculation shows that using an iEF of 3 in the case of acetaldehyde would lead to a rather conservative OEL as compared to using a PBPK model.

*Ammonia* A human experimental study revealed increased symptom ratings and the perception of an unpleasant odor at 25 ppm but no objective signs of irritative effects (NOAEC) (Sundblad et al. 2004). The application of an iEF of 3 would give a concentration of 75 ppm. Additional adjustment for time extrapolation by application of a tEF of 2 for subchronic effects results in a theoretical animal NOAEC of 150 ppm. From an animal study with continuous exposure (24 h/day, 35–49 days) (Broderson et al. 1976), a NOAEC of 200 ppm (LOAEC 250 ppm) was derived in rats. This is in accordance with the theoretical NOAEC derived from the human data although one needs to consider that the exposure regimen in the animal study (continuous exposure over 5–7 weeks) is quite unusual and creates some uncertainty.

According to recent studies (DFG 2000; Hoffmann et al. 2004a, b; Ihrig et al. 2006) even at 50 ppm, strong irritation effects do not seem to occur.

*n*-*Butyl acetate* In a human experimental study, very slight irritative effects—according to the authors not to be considered as adverse—and the perception of an unpleasant odor were reported at 147 ppm for 4 h (NOAEC) and 295 ppm for 20 min; higher concentrations were not tested (Iregren et al. 1993). Application of the iEF of 3 to the NOAEC of the longest duration from this human study would lead to a NOAEC of 441 ppm. Additional time extrapolation for a theoretical subchronic animal NOAEC with a tEF of 2 would lead to 882 ppm. From a subchronic inhalation study in rats (6 h/day, 5 days/week, 14 weeks), a NOAEC of 500 ppm and a LOAEC of 1,500 ppm were derived. Minimal to mild degeneration of the OE along the dorsal medial meatus and ethmoturbinates of the nasal passages was seen at 1,500 ppm and more pronounced at 3,000 ppm (David et al. 1998, 2001). Since the dosage in the rat inhalation study covered the range from 500 to 1,500 ppm as LOAEC, the “real” concentration without effect NAEC can be regarded as consistent with the 882 ppm derived from the corresponding human study.

*2*-*Ethylhexanol* A controlled human study that found moderate to strong odor intensity and increased substance P in nasal lavage, indicating neurogenic inflammation, resulted in a human LOAEC of 20 ppm (van Thriel et al. 2007). Based on the calculation of a benchmark dose analysis for eyeblink frequency, a human NOAEC of 15 ppm was estimated (DFG 2012). Application of an iEF of 3 would give a theoretical NOAEC for a chronic animal study of 45 ppm. Time extrapolation would lead to theoretical NOAECs of 90 ppm for a subchronic and 270 ppm for a subacute animal study. In a subacute (6 h/day, 5 days/week, 2 weeks) and a subchronic (6 h/day, 5 days/week, 12 weeks) animal study, the highest concentration tested was 120 ppm (BASF 1992). No adverse effects were found at this concentration, so that 120 ppm was defined as NOAEC. A LOAEC was not obtained. Therefore, dose response relation had not been fully elaborated, and with this limitation, this comparison includes some uncertainties. Nevertheless, if combined with an iEF of 3 the results still appear to be compatible with the value derived from the human study.

*Hydrogen sulfide* A controlled human study which included ratings of acute symptoms and various aspects of chemosensory perception, postural sway, contrast sensitivity, and cognitive performance yielded a human NOAEC of 5 ppm (Fiedler et al. 2008). An iEF of 3 would give a concentration of 15 ppm. As the daily exposure duration in the subacute study was only 3 h/day, an adjustment via the time-concentration product is necessary to extrapolate a NOAEC for 8/day. Thus, a subacute NAEC of 10 ppm was calculated which is the same as the NOAEC of the subchronic study, and therefore, time extrapolation is not necessary (OEL Documentation “hydrogen sulfide,” TRGS 900, http://www.baua.de/de/Themen-von-A-Z/Gefahrstoffe/TRGS/pdf/900/900-schwefelwasserstoff.pdf?__blob=publicationFile&v=2). This would lead to theoretical NOAEC of 15 ppm for a subchronic and 15 ppm for a subacute animal study. However, the real NOAEC from the subchronic study is 10 ppm and the LOAEC 30 ppm for effects in the OE of rats (Brenneman et al. 2000; Dorman et al. 2004). The application of an iEF of 3 is therefore in line with the experimental results in animals. Moreover, a reduction in the default factor of 3 is warranted in that case, because the target tissue is OE and there are modeling data on the extraction of hydrogen sulfide in the nose of rats and humans (OEL Documentation “hydrogen sulfide,” TRGS 900, http://www.baua.de/de/Themen-von-A-Z/Gefahrstoffe/TRGS/pdf/900/900-schwefelwasserstoff.pdf?_blob=publicationFile&v=2). These data predict that the subchronic NOAEC of 10 ppm corresponds to 21 ppm for humans.

In conclusion, the comparisons of the theoretical NOAECs derived from human data with application of the iEF of 3 with the available data from subacute and subchronic animal studies demonstrate that under conditions of standard tEFs, no significant discrepancies for four of the five compounds. Only in the case of 2-ethylhexanol would the human NOAEC (15 ppm) be slightly overpredicted (theoretical human NOAEC 20 ppm) with the application of an iEF of 3 to the animal data.

The default iEF“3” was also evaluated for the group of compounds with lower data density:

*n*-*Butyl amine* A controlled human study is lacking, and the human NOAEC of 2 ppm from exposed workers is not well documented (Beard and Noe 1981). An iEF of 3 would arrive at a concentration of 6 ppm. This would lead to a theoretical NOAEC of 108 ppm for a theoretical subacute LOAEC from an animal study (1/3 NOAEC/LOAEC; 1/6 subacute to chronic). Actually a LOAEC of 17 ppm was found in a subacute study in rats (14 days) (BASF 2001).

*Chlorine* The human NOAEC of 0.5 ppm is based on experimental studies (e.g., Rotman et al. 1983; Schins et al. 2000) which indicated no significant changes in lung function or signs of inflammation effects in the nose up to this concentration. An iEF of 3 leads to 1.5 ppm for the NOAEC from a theoretical chronic animal study and to a LOAEC of 4.5 ppm.

A chronic LOAEC of 0.4 ppm was derived from a chronic inhalation study in rats and mice (6 h/day, 5 days/week, 24 months), which revealed hypertrophy of goblet cells in rats and hyperplasia in the respiratory epithelium in mice at this dosage (Wolf et al. 1995).

*Methyl acetate* No irritation in volunteers of a toxicokinetic study exposed twice/day for 2 h each was reported up to 200 ppm methyl acetate. A LOAEC was not available (Tada et al. 1974). Five minutes of exposure toward 325 ppm methyl acetate were tolerated (NOAEC), whereas 4,050 ppm (LOAEC) irritated trachea and throat (Flury and Wirth 1933). This study did not meet the inclusion criteria of at least 2 h of exposure. The theoretical subacute NOAEC starting from the NOAEC of 200 ppm would be 3,600 ppm (iEF 3, tEF 6). From a subacute inhalation study in the rat (6 h/day, 5 days/week, 28 days), no effects were seen at the lowest dose of 350 ppm (NOAEC) and degeneration and necrosis of the OE at 2,000 ppm (Celanese 1999).

*Vinyl acetate* A controlled human study is not available but a human NOAEC of 10 ppm (LOAEC 22 ppm) was based on insufficiently documented observational data (Deese and Joyner 1969). Application of the extrapolation factor 3 for interspecies extrapolation to the human NOAEC results in 30 ppm. Though the quality of these data is poor, they are in quite good agreement with the NOAEC of 50 ppm (LOAEC 200 ppm) which was found in a chronic inhalation study (6 h/day, 5 days/week, 24 months) in rats and mice. Adverse effects that were seen at and above the LOAEC of 200 ppm were atrophy, regenerative processes, inflammation, and metaplasia in the OE as well as basal cell hyperplasia (Bogdanffy et al. 1994).

Application of the default factor “3” to the group of compounds with lower data density does not necessarily arrive at values obtained experimentally. In turn, however, application of the iEF of 3 will definitely not underestimate human risk. Thus, it can be concluded that the iEF of 3 can obviously be used generally as default factor.

During the assessment process, it became clear that the remaining seven compounds dimethyl sulfoxide, dipropylene glycol, glycerol, methoxyacetic acid, 4-methyl-3-penten-2-on, pentanol isomers, trimethylamine did not meet the criteria for consideration in this paper. For example, in the case of methoxyacetic acid, systemic effects are more sensitive. Glycerol is another example in which irritation was observed but not as the most sensitive end point. Results from animal studies with glycerol show that direct application to the animal eye results only in a very slightly irritating effect. A chronic local effect at a different location outside the URT (squamous cell metaplasia at the epiglottis) was considered the most sensitive end point (DFG 2006).

## Conclusions

Comparison of human data with data from subacute and subchronic animal studies for those compounds with the most complete database lead us to conclude that an iEF of 3 is the most reasonable factor for extrapolating animal data concerning local irritating effects.

**Fig. 4:**
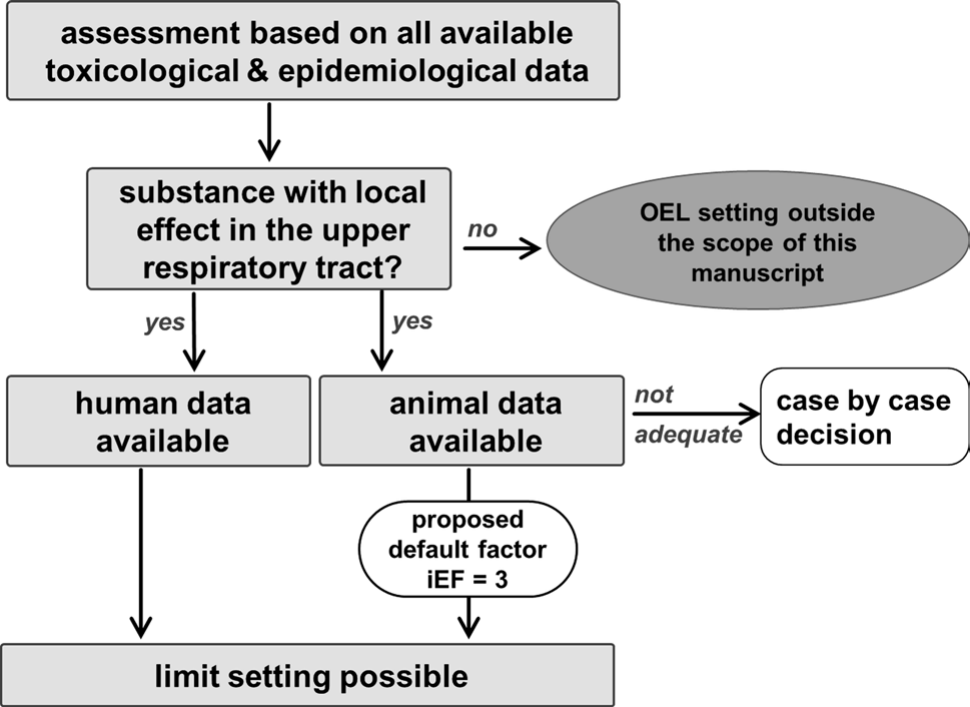
An idealized scheme for the procedure suggested for setting an OEL from data regarding sensory irritation

This was confirmed by the application of this factor to additional compounds with lower data density. Thus, we propose that an iEF of 3 should be applied for extrapolation from animal data for all those substances with indication of local irritating effects as the most sensitive response (“leading health effect”) in animal studies but without reliable human data; unless individual data argue against this approach. In such cases, a substance-specific approach should be applied (Fig. 4). The example of hydrogen sulfide shows how substance-specific data on dosimetric species differences can be used to refine the risk assessment.

In case the target structure in the animal experiment is the OE, it should be considered to reduce the default iEF to 2 because modeling of the airways of rats and humans have shown that in humans the fraction of the inhaled air reaching the OE is only half of that in rats (Frederick et al. 1998).

Due to the intense and ongoing discussion about how good human data can be generated to serve OEL setting in the context of sensory irritation (e.g., van Thriel et al. 2006a), this idealized scheme might be applicable now and in the future. However, if only human studies of minor quality are available (see “Part 2: Local effects of irritants on the upper respiratory tract and the mucous membranes of the eyes in the working environment—sensory and non-sensory origins” section; definition of a sensory NOAEC_human_), expert judgment is necessary to evaluate whether the flaws of the study preclude the derivation of a point of departure. Guidance for this evaluation is given in several sections in this overview.

### Interindividual differences in chemosensory-mediated effects

Regarding interindividual differences in humans, equivalent to an *intraspecies* default factor, there are not many studies available that systematically investigated the role of such factors on chemosensory-mediated effects. Taking into account non-clinical differences (e.g., self-reported chemical sensitivity), interindividual differences might contribute little to chemosensory-mediated effects in a healthy population.

Various studies investigating lateralization thresholds (Dalton et al. 2000; van Thriel et al. 2006a) or other psychophysical techniques (Cometto-Muniz and Cain 1990, 1998) to estimate the trigeminal potency of a certain chemical reported large interindividual differences in trigeminal and olfactory sensitivity. Such information can hardly be used in the context of trigeminal-mediated defense mechanism that are the first adverse effects according to the model proposed in Fig. 3. In a series of experiments, the IfADo Lab investigated the role of sCS on different chemosensory effects [e.g., breathing frequency, eyeblink frequency (EBF), subjective ratings]. In these “sensitive” individuals, the breathing frequency was only slightly higher at the beginning of exposures to ethyl benzene or 2-butanone, both applied in OEL concentrations (Haumann et al. 2003), but decreased during the 4-h exposure. However, in a second experiment investigating 2-propanol and 1-octanol, these interindividual differences could not be observed. When evaluating chemosensory effects of acute exposures to 2-ethylhexanol (up to 40 ppm during exposure peaks), sCS and control subjects yielded comparable eyeblink frequencies (Kiesswetter et al. 2005). Only at the beginning of constant exposures to 1.5, 10, or 20 ppm, the sCS subjects had higher EBFs. However, these interindividual differences were not exposure-related. When evaluating the impact of sCS on different psychometric ratings of the intensity of chemosensory perceptions or chemosensory-mediated health symptoms, only the latter were slightly increased in the sCS group (van Thriel et al. 2005). Another group investigated the impact of CO_2_ sensitivity as a marker of trigeminal sensitivity in the context of experimental exposures to formaldehyde (Mueller et al. 2013). No differences between hypo- and hypersensitive subjects were found for physiological measures of chemosensory-mediated effects (e.g., EBFs) during various exposures to formaldehyde (including exposure peaks of 0.8 ppm). A more clinical population was investigated by Shusterman et al. (2005). Patients with seasonal allergic rhinitis (SAR) were exposed to acetic acid and their responses were compared to non-allergic control subjects (Shusterman et al. 2005). When challenged with 15 ppm acetic acid for 15 min, SAR subjects showed a significant increase in nasal airway resistance (NAR; measured by active posterior rhinomanometry) than the control subjects. When normalized to baseline SAR subjects, NAR increased by 22 % while those of the controls decreased by 11 %. In a previous study, the same group showed similar differences for SAR subjects when challenged with chlorine (Shusterman et al. 2003). Patients with sensory hyperreactivity (Millqvist et al. 2008) showed not only a higher number of coughs when provoked with 0.4 or 2 µM of capsaicin aerosols, their response was dose-dependent and increased by pre-inhalation of ethanol (5 and 25 %). Control subjects showed no response to ethanol inhalation.

In conclusion, patients suffering from SAR, sensory hyperreactivity, or asthma (Roger et al. 1985) might show stronger chemosensory-mediated effects to local irritants than healthy controls. However, effects in asthmatics might be chemical dependent since ammonia did not provoke stronger effects in these patients (Petrova et al. 2008).

It is suggested that an intraspecies default factor is not necessary if OELs are derived from human sensory NOAECs since it is based on a controlled human exposure study assessing especially sensitive and objectively verifiable effects. Particularly, “sensitive” individuals can be considered by the choice of the adequate statistical methods. Like for other toxicological end points, benchmark concentration levels (BMCLs) might be derived to account for intraspecies differences. In toxicological risk assessment, this approach was used for setting the OEL of 2-ethylhexanol in the current MAK value documentation (DFG 2012). More general, this approach was used for the evaluation of acute SO_2_ effects on breathing depth in humans (Kleinbeck et al. 2011). Hence, the evaluation method always depends on study design and has to be selected accordingly. Moreover, human studies could include healthy volunteers, who were identified as “sensory sensitive” with the help of specific tests (e.g., CO_2_ test, capsaicin test, sCS questionnaire). So far, available data indicate that an intraspecies default factor >1 is not necessary whenever good experimental exposure studies with human volunteers are available.

### A tentative OEL based on RD_50_ values

Some authors (e.g., Schaper 1993) proposed a conversion factor of 0.03 between the RD_50_ in mice, and a tentative OEL. This approach, which is quite schematic, does not take into account repeated dose toxicity and different biological mechanisms of different compounds. Cytotoxic and tissue damaging effects for example are not well predicted by RD_50_ values (Bos et al. 2002). Therefore, RD_50_ value should be limited to an indicative tool for those compounds which might exert a sensory irritation below the thresholds for observable morphological effects. The basis for the OELs used in this correlational approach might be (a) human exposure studies addressing chemosensory effects or (b) inhalational studies in animals measuring tissue irritation and OELs should not be based solely on this value. It might be useful when comparing structurally similar compounds (e.g., aliphatic amines) with a common mode of action. Moreover, the detailed analysis of breathing patterns in mice can provide valuable information to distinguish sensory irritation of the URT from airflow obstruction/limitations and pulmonary irritation (e.g., Boylstein et al. 1996; Alarie 1998). Based on this approach, substances that might affect LRT without causing sensory irritation can be identified.

### Odor perception

Assessment of adverse effects occurring from odor must be dealt separately from irritation. This is due to the fact that such effects are only observed directly in humans. Further, it is unclear whether interindividual variability factors need to be considered for these compounds as their adversity occurs on a very individual level.

It is worth noting that OSHA has regulated three chemicals based on adverse odor effects: isopropyl ether, phenyl ether, and vinyl toluene (OSHA 1989). These limit values were set based on worker complaints and the assumption that these substances can cause distraction effects creating safety hazards (van Thriel et al. 2003).

Even though it is still difficult to assess the adversity of odor effects at least, they should be taken into account as additional information. For instance, odor perception is a warning signal and adaptation itself could be considered an adverse effect in situations where irritation is severe and a substance’s odor may warn of the substance’s presence before irritating effects occur (Paustenbach and Gaffney 2005).
